# Detection of Acute HIV-1 Infection by RT-LAMP

**DOI:** 10.1371/journal.pone.0126609

**Published:** 2015-05-20

**Authors:** Donna L. Rudolph, Vickie Sullivan, S. Michele Owen, Kelly A. Curtis

**Affiliations:** Laboratory Branch, Division of HIV/AIDS Prevention, National Center for HIV/AIDS, Hepatitis, STD, and TB Prevention, Centers for Disease Control and Prevention, Atlanta, GA, United States of America; Centro Nacional de Microbiología - Instituto de Salud Carlos III, SPAIN

## Abstract

A rapid, cost-effective diagnostic test for the detection of acute HIV-1 infection is highly desired. Isothermal amplification techniques, such as reverse-transcription loop-mediated isothermal amplification (RT-LAMP), exhibit characteristics that are ideal for the development of a rapid nucleic acid amplification test (NAAT) because they are quick, easy to perform and do not require complex, dedicated equipment and laboratory space. In this study, we assessed the ability of the HIV-1 RT-LAMP assay to detect acute HIV infection as compared to a representative rapid antibody test and several FDA-approved laboratory-based assays. The HIV-1 RT-LAMP assay detected seroconverting individuals one to three weeks earlier than a rapid HIV antibody test and up to two weeks earlier than a lab-based antigen/antibody (Ag/Ab) combo enzyme immunoassay (EIA). RT-LAMP was not as sensitive as a lab-based qualitative RNA assay, which could be attributed to the significantly smaller nucleic acid input volume. To our knowledge, this is the first demonstration of detecting acute HIV infection using the RT-LAMP assay. The availability of a rapid NAAT, such as the HIV-1 RT-LAMP assay, at the point of care (POC) or in laboratories that do not have access to large platform NAAT could increase the percentage of individuals who receive an acute HIV infection status or confirmation of their HIV status, while immediately linking them to counseling and medical care. In addition, early knowledge of HIV status could lead to reduced high-risk behavior at a time when individuals are at a higher risk for transmitting the virus.

## Introduction

Routine diagnostic testing is imperative for the early detection and treatment of HIV infection. Because individuals are at higher risk for transmitting the virus during early or acute infection, accurate and timely diagnosis may reduce the transmission of HIV when the individual is most infectious [1]. Early detection of HIV has been shown to lead to reduced high-risk behavior and to connect individuals to treatment earlier, which can reduce the risk of virus transmission [2, 3]. In 2006, an MMWR was published that advocated routine, voluntary testing of adults, adolescents, and pregnant women aged 13–64 years in health-care settings as normal practice [2]. Although there are currently a large number of FDA-approved HIV diagnostic tests with high sensitivities and specificities available, there were still 1.1 million people in the U.S. living with HIV at the end of 2011, of which, 15.8% remained undiagnosed or were unaware of their infection status [4]. Point-of-care (POC) testing has increased the number of individuals who are screened for HIV and receive their HIV test results [5]. In the U.S., examples of POC settings may include, but are not limited to, clinics, mobile testing units, jails, and emergency rooms.

For laboratory settings, a revised HIV testing algorithm has been published to improve upon the accurate detection of acute HIV-1 infection, as well as HIV-2 [6]. In this algorithm, specimens are screened with a sensitive HIV-1/2 immunoassay, preferably a fourth-generation antigen/antibody assay, followed by an HIV-1/2 differentiation assay. Specimens that are non-reactive are considered negative. Specimens that have concordant reactivity on the screening and supplemental test are considered positive for HIV-1/2 antibodies; however, in the case of discordant immunoassay results, HIV-1 nucleic acid amplification testing (NAAT) is recommended. NAAT is highly sensitive, virus specific, and allows for detection of infection approximately two weeks earlier than most antibody-based tests [5, 6].

To date, there are no definitive guidelines for HIV testing at the POC. Rapid tests have facilitated HIV testing at the POC because they can be completed in a short period of time (typically less than 30 minutes) and require minimal technical expertise. Currently, there are a number of rapid antibody tests available that are FDA-approved; however, they are not as sensitive for detection of early HIV infection as most laboratory based assays and will remain negative during the period post-infection, but pre-seroconversion [7]. The availability of a rapid NAAT for use at the POC could increase the ability to detect acute infection. The Aptima HIV-1 Assay (Hologic Inc., San Diego, CA) is currently the only FDA-approved diagnostic NAAT, but its use is not feasible for the POC due to the high cost per test, dedicated equipment requirements, and the need for trained technical staff. Ideally, a rapid NAAT should be completed in a short time frame with a few simple steps, be easy to interpret, and require no or minimal equipment. In addition, the rapid NAAT must exhibit a high degree of sensitivity and specificity. Isothermal amplification techniques are attractive for the development of a rapid NAAT because they do not require thermal cycling and, therefore, the reaction can be run in a simple heat block, water bath, or other portable heating device [8]. One such isothermal technique, loop-mediated isothermal amplification (LAMP), has been developed for the detection of DNA [9] and RNA (reverse-transcription, loop-mediated isothermal amplification or RT-LAMP) [10–13].

The LAMP technique has several characteristics that are appealing for the development of a rapid NAAT. The amplification method is highly specific because it requires six primers that recognize eight different sequences in the same target region. The method is less sensitive to biological inhibitors than PCR, which allows for amplification directly from biological samples, such as whole blood, plasma and oral fluid, without the need for extraction of nucleic acid [14–18]. Amplified material can be detected within 15–60 minutes when incubated at a constant temperature (60–65°C) and immediate visual detection is possible due to the large amount of DNA generated from each reaction [19]. In addition, several groups have incorporated fluorescent detection methods into the LAMP assay for real-time or immediate naked-eye detection [17] [19–22]. We have demonstrated previously the development of an HIV-1 RT-LAMP assay that incorporates a sequence-specific detection method and allows for the simultaneous detection of both RNA and DNA in a single reaction tube [17].

In this study, we evaluated the ability of the HIV-1 RT-LAMP assay to detect acute HIV infection as compared to a representative rapid antibody test and several laboratory-based assays. Using two primer sets directed against highly conserved regions within the reverse transcriptase (RT) and integrase (INT) genes and well-characterized seroconversion panels from recent seroconverters, we demonstrated that the HIV-1 RT-LAMP assay was able to detect samples from acutely infected individuals up to 24 days prior to a representative rapid antibody test. The HIV-1 RT-LAMP assay, used in conjunction with current rapid antibody tests, has the potential to increase the number of individuals who receive an accurate and timely HIV result at the POC or in select laboratory settings, where cost and time restraints prohibit the use of standard NAAT platforms.

## Materials and Methods

### RT-LAMP Primers and Quenchers

HIV-1 RT-LAMP primers specific for the RT gene region have previously been described [8]. The RT primers recognize target sequences within the genome location 2900–3118, relative to the HXB2 reference strain. In this study, a truncated version of the RT quencher probe with a 3’ BHQ-1 label (AAACAATGAGACACC-BHQ1) was designed to be added directly into the reaction mix as opposed to adding post-reaction, as in previous studies [8, 17]. This eliminates the need to open the reaction tubes after amplification and reduces the risk of amplicons being released and contaminating the work environment. An additional HIV-1 primer set was designed in a highly conserved region of the INT gene, using PrimerExplorer V3 software available on the Eiken Chemical Co. Ltd. (Japan) website (http://primerexplorer.jp/e/). A region within the integrase gene was selected for primer design due to the high degree of sequence conservation within subtype B and across the group M subtypes, as summarized in the HIV Sequence Compendium (http://www.hiv.lanl.gov/). The HIV-1 HXB2 sequence (GenBank accession number AF033819) was used as the reference for generating the primers. The sequences of the INT primers and quencher probe are listed in Table 1. All primers were synthesized in-house and the FIP and BIP primers were HPLC purified.

**Table 1:** INT Primer and Quencher Probe Sequences.

Primer Name	HXB2 Location	Sequence (5’ → 3’)
F3	4901–4920	GGTTTATTACAGGGACAGCA
B3	5070–5087	ATCCTGTCTACTTGCCAC
LoopF	4943–4962	CTTTCCAGAGAAGCTTTGCT
LoopB-HEX	5021–5042	HEX-AGCAAAGATCATTAGGGATTAT
FIP	4963–4986, 4923–4942	CTTGTATTACTACTGCCCCTTCACGATCCACTTTGGAAAGGACC
BIP	4994–5018, 5051–5069	TGACATAAAAGTAGTGCCAAGAAGATTTTACAATCATCACCTGCCATC
LoopB-Q	5021–5034	TAATGATCTTTGCT-BHQ1

### HIV-1 DNA and RNA Linearity Panels

An HIV-1 DNA linearity panel was created using the human monocytic cell line OM-10.1 [23], which contains a single integrated HIV-1 provirus per cell. DNA was extracted using a QIAamp DNA Blood Mini kit (Qiagen, Valencia, CA), as previously described [17]. A panel was created ranging from 10^5^ to 10^2^ copies/ml using serial, tenfold dilutions in diethylpyrocarbonate (DEPC)-treated water (Invitrogen, Carlsbad, CA). A negative DNA control was generated by extracting DNA from peripheral blood mononuclear cells (PBMCs) infected with an HIV-2 isolate, NIH-Z (Advanced Biotechnologies, Inc., Columbia, MD). Due to the genetic diversity between HIV-1 and HIV-2 and the specificity of the RT-LAMP assay, HIV-1 primers do not amplify HIV-2 targets.

An HIV-1 RNA linearity panel was created using the supernatant from 8E5 cells (ATCC, Manassas, VA), which contain a single defective proviral genome of HIV-1 per cell, but still express virus in the supernatant. The viral load of the cell supernatant was determined using the Roche COBAS AmpliPrep / COBAS TaqMan HIV-1 Test V2.0 (Roche Diagnostics, Indianapolis, IN) and the supernatant was spiked into HIV-1 seronegative human plasma. Serial dilutions were made in plasma to create a panel that ranged from 10^6^ to 10^3^ RNA copies/ ml. RNA was extracted from the panel using a QIAamp Viral RNA Mini Kit (QIAGEN, Valencia, CA). Positive and negative controls included RNA extracted from HIV-1 BaL purified virus and HIV-2 NIH-Z purified virus (Advanced Biotechnologies Inc., Columbia, MD), respectively. Aliquots of both linearity panels were made and stored at -80°C until use.

### HIV-1 Seroconversion Panels

Serial plasma specimens were collected from 12 US donors who became HIV-1 infected during the collection period. The seroconversion panels were obtained from SeraCare Life Sciences (n = 7) (Gaithersburg, MD) and ZeptoMetrix Corp. (n = 5) (Buffalo, NY) [24, 25]. The Multispot HIV-1/HIV-2 Rapid Test (Bio-Rad, Redmond, CA), GS HIV-1 Western blot (Bio-Rad), third-generation GS HIV-1/HIV-2 Plus O EIA (Bio-Rad), fourth-generation GS HIV Combo Ag/Ab EIA (Bio-Rad), and APTIMA HIV-1 Qualitative Assay (Hologic Inc., San Diego, CA) test results were compared with the results from the RT-LAMP assay. These test results are represented as rapid Ab, WB, Ab EIA, Ag + Ab EIA and qualitative RNA, respectively. For the SeraCare panels, all HIV test results were supplied by the manufacturer, with the exception of the Multispot and the APTIMA, which were performed in-house according to the package inserts. Viral loads were supplied by the manufacturer and were determined using the COBAS AmpliPrep / COBAS TaqMan HIV-1 Test V2.0 (Roche Diagnostics, Indianapolis, IN), except for panels PRB970 and PRB946, which were determined by the COBAS AmpliPrep / COBAS TaqMan HIV-1 Test V1.0 (Roche Diagnostics, Indianapolis, IN) and the Amplicor HIV-1 Monitor Test (Roche Diagnostics, Indianapolis, IN), respectively. The lower limit of detection of the COBAS TaqMan HIV-1 Test V2.0, V1.0, and Amplicor HIV-1 Monitor Test is 20, 40, and 50 copies/ mL, respectively. For the Zeptometrix panels, all HIV testing was performed in-house according to the package inserts. The viral loads for each sample were determined by the COBAS AmpliPrep / COBAS TaqMan HIV-1 Test V2.0. For RT-LAMP testing, RNA was extracted from the panels using a QIAamp Viral RNA Mini Kit (Qiagen, Valencia, CA).

### RT-LAMP Reaction

The RT-LAMP reaction was performed as described previously, with a few modifications [17]. Briefly, the RT-LAMP reaction mix (25 μL volume total) contained a final concentration of 0.2 μM each of F3 and B3 primers, 1.6 μM each of FIP and BIP primers (both HPLC purified, as recommended [26]), 0.8 μM each of LoopF and LoopB primers (a 5’ HEX label was added to LoopF for RT and LoopB for INT), 0.8M Betaine (Sigma-Aldrich, St. Louis, MO), 8 mM MgSO_4_, 1.4 mM dNTPs (Roche Applied Science, Indianapolis, IN), 1X Isothermal Amplification Buffer (New England Biolabs, Ipswich, MA), 16U *Bst* 2.0 WarmStart DNA Polymerase (New England Biolabs) and 2U AMV Reverse Transcriptase (Life Technologies, Carlsbad, CA). The addition of a reverse transcriptase enzyme allows the amplification of both DNA and RNA simultaneously in the same reaction tube. In addition, a 3’ BHQ1-labeled quencher probe was added into the reaction at a final concentration of 0.8μM. To the reaction mixture, 10 μL of extracted DNA or RNA was added. The reaction mixture was heated at 60°C for 60 minutes, using a GeneAmp PCR System 9700 (Applied Biosystems, Foster City, CA), then held at 80°C for 2 minutes to terminate the reaction. For the purposes of the current evaluation, each specimen was tested in duplicate with each primer set. In addition to positive and negative controls, a reagent (no template) control was included in every run to check for reagent contamination. The presence of amplified product was determined visually by observing fluorescence in the reaction tubes using the UV lamp from a GelDoc XR+ Imaging System (BioRad Laboratories, Hercules, CA).

## Results

### HIV-1 INT RT-LAMP Sensitivity

**Fig 1:**
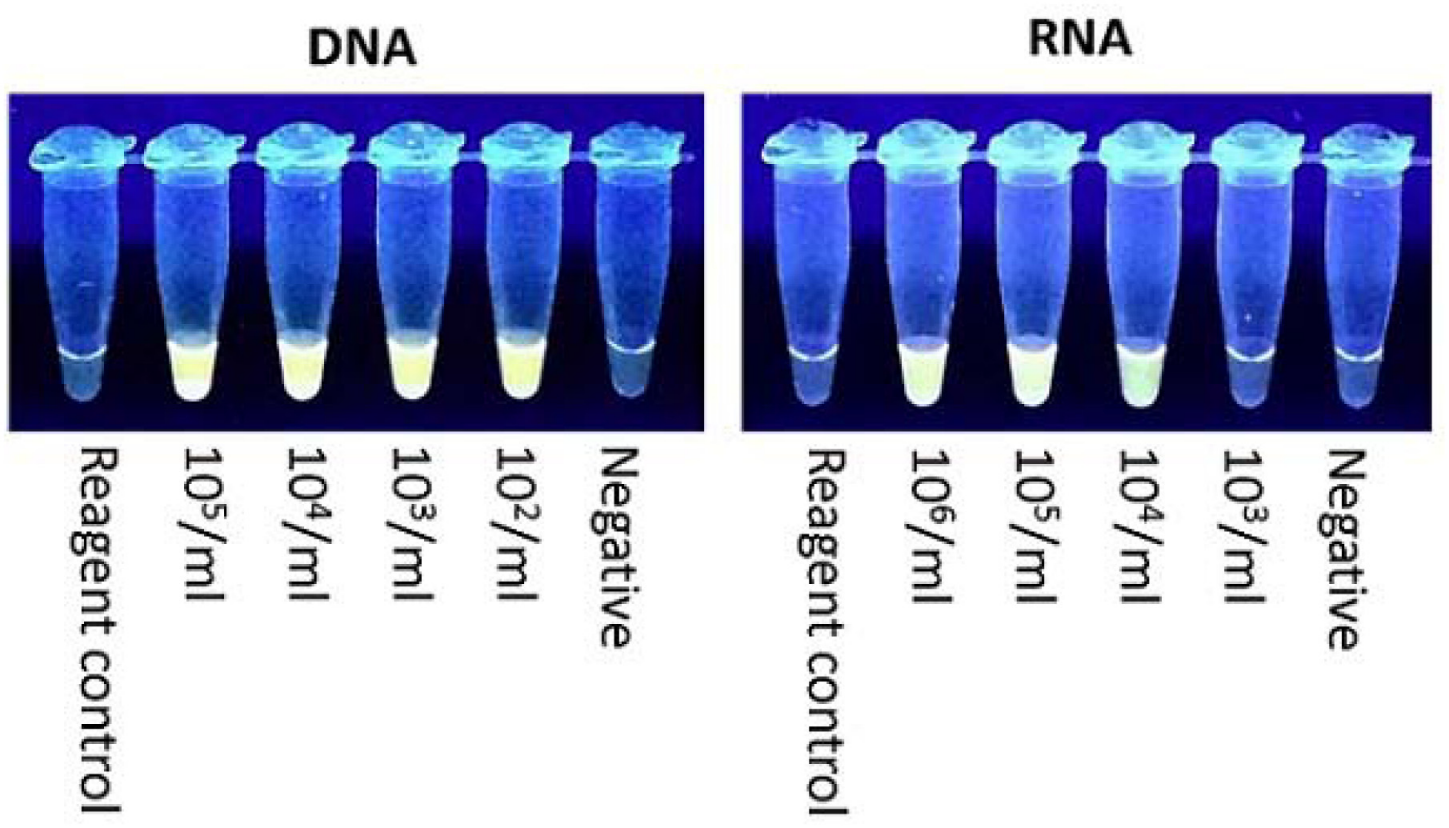
HIV-1 INT RT-LAMP Sensitivity. The RT-LAMP reaction tubes were observed under UV light. Representative tubes for amplification of DNA and RNA are shown.

The sensitivity of the HIV-1 RT-LAMP assay using the RT primers has been previously described [8]. The sensitivity of the assay for both DNA and RNA using the INT primers was similar to the reported sensitivity for the RT primers. A representative experiment demonstrating amplification of HIV-1 DNA and RNA linearity panels is shown in Fig 1. The limit of detection for DNA was 10^2^ copies/ mL and 10^5^–10^4^ RNA copies/ mL, depending on the experiment. No amplification was observed in the negative control or the reagent control tube.

### Evaluation of RT-LAMP Assay with Acute HIV-1 Clinical Samples

To determine the ability of the RT-LAMP assay to detect samples from acute HIV-1 infection, seroconversion panels were tested. For the SeraCare samples (n = 7), only one of the panels became reactive during the sample collection period by the rapid Ab test. This particular sample became rapid Ab reactive 14 days after the first detectable viral load and the first qualitative RNA positive (Table 2). In contrast, all of the SeraCare panels were reactive by the HIV-1 RT-LAMP assay between 0 and 2 days (median = 0) after the first detectable viral load and qualitative RNA positive. For most panels, both the RT and INT primers detected samples from the same time point; however, in select cases, one primer set detected a sample prior to the other. Based on the viral load data from the panel, the RT primer set detected all of the SeraCare samples in the expected sensitivity range of the assay (> 10^4^ RNA copies/ mL) and the INT primer set detected 6 out of 7 of the panels in the expected sensitivity range. In comparison to the laboratory-based tests, the RT-LAMP assay detected members in all seroconversion panels prior to the Ab EIA, and 0 to 7 days (median = 3.5) prior to the Ag + Ab EIA. None of the seroconversion panel members became WB positive during the sample collection period.

**Table 2:** Evaluation of RT-LAMP Assay with SeraCare Seroconversion Panels.

Panel ID	Days Since 1^st^ VL	Viral Load	Qual RNA	RT RT-LAMP	INT RT-LAMP	Ag+Ab EIA	Ab EIA	WB	Rapid Ab
PRB973-01	0	1.8x10^3^	+	-	-	-	-	-	-
PRB973-02	2	1.7x10^4^	+	+	+	-	-	-	-
PRB973-03	7	1.7x10^5^	+	+	+	+	-	-	-
PRB973-04	11	1.5x10^6^	+	+	+	+	+	IND	-
PRB974-01	-7	BLD	-	-	-	-	-	-	-
PRB974-02	0	8.6x10^3^	+	+	+	-	-	-	-
PRB974-03	2	8.3x10^4^	+	+	+	+	-	-	-
PRB974-04	9	9.6x10^5^	+	+	+	+	-	-	-
PRB975-01	-7	BLD	-	-	-	-	-	-	-
PRB975-02	-5	BLD	-	-	-	-	-	-	-
PRB975-03	0	1.4x10^2^	+	-	-	-	-	-	-
PRB975-04	2	2.1x10^3^	+	+	+	-	-	-	-
PRB975-05	7	1.8x10^6^	+	+	+	+	-	-	-
PRB976-01	0	1.2x10^4^	+	+/-	+	-	-	-	-
PRB976-02	2	2.1x10^4^	+	+	+	-	-	-	-
PRB976-03	7	6.3x10^5^	+	+	+	+	-	-	-
PRB976-04	9	1.9x10^6^	+	+	+	+	-	-	-
PRB977-01	0	1.9x10^2^	+	-	-	-	-	-	-
PRB977-02	2	3.5x10^3^	+	-	+/-	-	-	-	-
PRB977-03	13	1.6x10^6^	+	+	+	+	-	-	-
PRB977-04	15	1.0x10^7^	+	+	+	+	+	-	-
PRB970-01	0	1.6x10^5^	+	+	+	+	-	-	-
PRB970-02	7	>1x10^7^	+	+	+	+	-	-	-
PRB970-03	10	2.8x10^6^	+	+	+	+	+	-	-
PRB970-04	14	6.5x10^4^	+	+	+	+	+	IND	+
PRB946-01	-4	BLD	-	-	-	ND	-	-	-
PRB946-02	0	3.0x10^4^	+	+/-	-	ND	-	-	-
PRB946-03	3	7.0x10^5^	+	+	+	ND	-	-	-
PRB946-04	7	>8x10^5^	+	+	+	ND	-	-	-

+, reactive test result; -, non-reactive test result; +/-, reactive in 1 of 2 replicates; IND, indeterminate; ND, no data; *, first detectable viral load.

**Fig 2:**
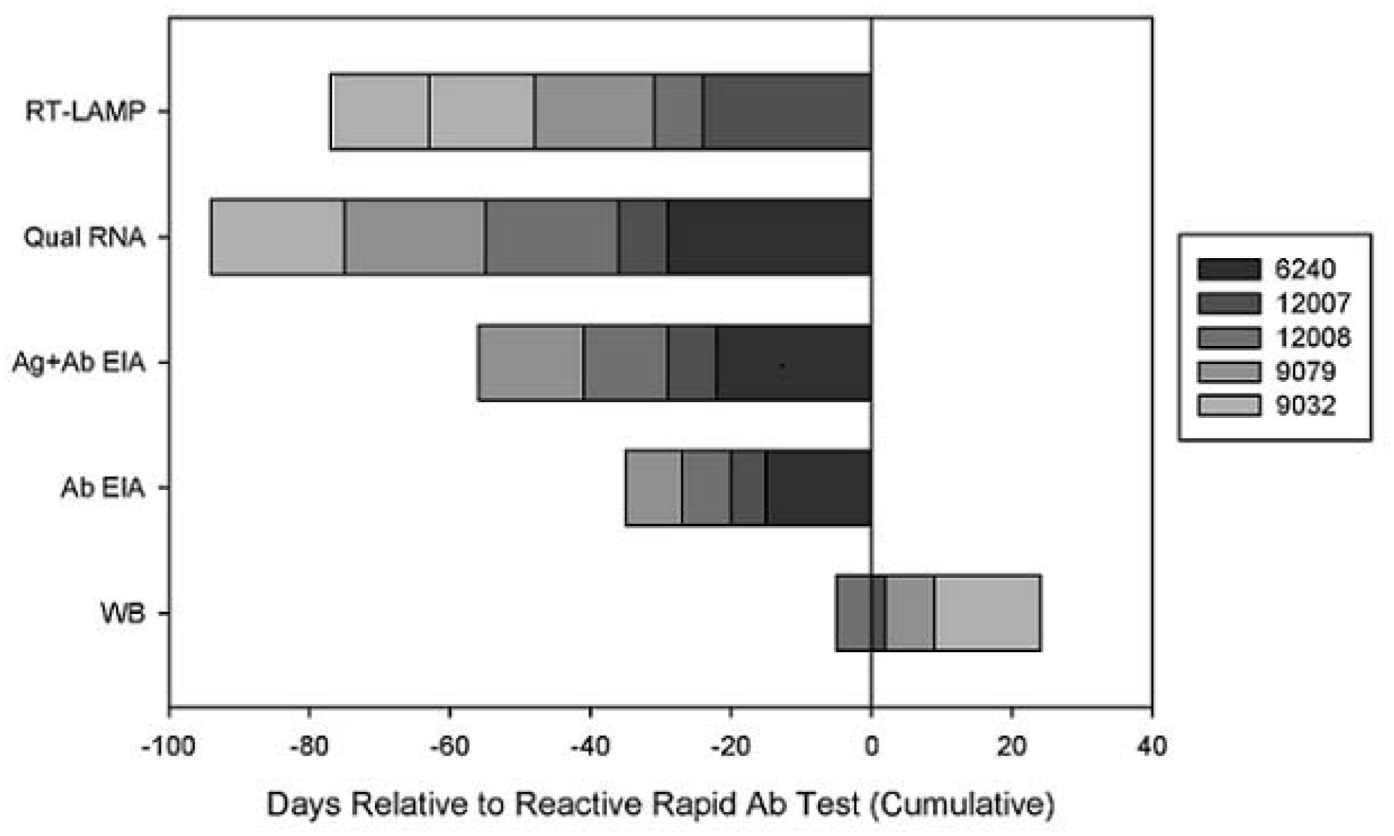
Summary of HIV-1 Test Results. The figure shows the timing of test positivity in relation to the rapid antibody test (Day 0) for the Zeptometrix cohort. Each segment of the stacked bar graph represents an individual subject.

For the ZeptoMetrix panels (n = 5), the rapid Ab test detected panel members 7 to 29 days (median = 19) post first detectable viral load and the first qualitative RNA positive (Table 3). In contrast, the RT-LAMP assay detected panel members within 0 to 5 days (median = 5) post first detectable viral load and qualitative RNA positive, which was 7 to 24 days (median = 15) earlier than the rapid Ab test. Overall, similar reactivity was observed between the RT and the INT primers for RT-LAMP with this panel. In comparison to the laboratory-based tests, the RT-LAMP assay detected panel members anywhere from 2 to 14 days (median = 9) prior to the Ab EIA and 0 to 14 days (median = 2) prior to the Ag + Ab EIA. The RT-LAMP assay was able to detect samples 9 to 29 days (median = 22) before the WB assay. Overall, the RT-LAMP assay detected all of the Zeptometrix panel members within our expected viral load sensitivity range (≥10^4^ RNA copies/ mL). A summary of the HIV-1 test results for these panels, in relation to the first rapid Ab reactive test result is shown in Fig 2. The SeraCare panels were not included in the timeline because of the short follow-up time and the fact that only one subject became rapid Ab test reactive during the sample collection period.

**Table 3:** Evaluation of RT-LAMP Assay with Zeptometrix Seroconversion Panels.

Panel ID	Days Since 1^st^ VL	Viral Load	Qual RNA	RT RT-LAMP	INT RT-LAMP	Ag+Ab EIA	Ab EIA	WB	Rapid Ab
6240–6	0	2.43x10^1^	+	-	-	-	-	-	-
6240–7	5	2.41 x 10^4^	+	+	+	-	-	-	-
6240–8	7	8.42 x 10^4^	+	+	+	+	-	IND	-
6240–9	12	7.28 x 10^5^	+	+	+	+	-	IND	-
6240–10	14	1.45 x 10^6^	+	+	+	+	+	IND	-
6240–12	29	5.07 x 10^4^	+	+	+	+	+	+	+
12007–3	-63	BLD	-	-	-	-	-	-	-
12007–4	0	1.80 x 10^5^	+	+	+	+	-	-	-
12007–5	2	1.29 x 10^5^	+	+	+	+	+	IND	-
12007–6	7	4.89 x 10^4^	+	+	+	+	+	IND	+
12007–7	9	4.91 x 10^4^	+	+	+	+	+	+	+
12008–6	-5	BLD	-	-	-	-	-	ND	-
12008–7	0	9.99 x 10^2^	+	+/-	-	-	-	ND	-
12008–8	2	3.20 x 10^4^	+	+	+	-	-	-	-
12008–9	7	4.70 x 10^6^	+	+	+	+	-	-	-
12008–10	12	> 1.0 x 10^7^	+	+	+	+	+	-	-
12008–11	14	> 1.0 x 10^7^	+	+	+	+	+	+	-
12008–12	19	1.09 x 10^5^	+	+	+	+	+	+	+
9079–8	0	2.08 x 10^1^	+	-	-	-	-	ND	-
9079–9	5	9.60 x 10^4^	+	+	+	+	-	-	-
9079–10	7	3.40 x 10^5^	+	+	+	+	-	-	-
9079–11	12	8.32 x 10^5^	+	+	+	+	+	-	-
9079–12	14	1.33 x 10^6^	+	+	+	+	+	IND	-
9079–13	20	1.56 x 10^5^	+	+	+	+	+	IND	+
9079–14	22	3.0 x 10^4^	+	+	+/-	+	+	IND	+
9079–15	27	7.21 x 10^3^	+	+	-	+	+	+	+
9032–6	0	1.62 x 10^3^	+	-	-	-	-	-	-
9032–7	5	4.49 x 10^4^	+	+	+	-	-	-	-
9032–8	7	2.76 x 10^4^	+	+	+/-	-	-	-	-
9032–9	12	2.81 x 10^4^	+	+	+	-	-	IND	-
9032–10	19	3.72 x 10^3^	+	+	+	+	+	IND	+
9032–11	21	3.42 x 10^3^	+	+	+	+	+	IND	+
9032–12	32	5.94 x 10^3^	+	+	-	+	+	IND	+
9032–13	34	1.03 x 10^3^	+	+/-	-	+	+	+	+

+, reactive test result; -, non-reactive test result; +/-, reactive in 1 of 2 replicates; IND, indeterminate; ND, no data; *, first detectable viral load.

## Discussion

The primary goal of this study was to demonstrate the utility of the HIV-1 RT-LAMP assay for detection of acute HIV-1 infection. To our knowledge, this is the first demonstration of acute HIV infection detection using the RT-LAMP assay. In previous studies, we demonstrated the usefulness of the HIV-1 RT-LAMP assay for detection of both DNA and RNA in a single reaction [16, 17]. In the current study, the primary focus was RNA detection, since high plasma viral loads are characteristic of acute infection; however, proviral DNA may also be detected in whole blood. To enable immediate naked eye visualization of the amplified products, a sequence-specific fluorescent detection method was developed for the HIV-1 RT-LAMP assay [17]. We further modified the detection method by adding the quencher probe directly into the reaction, which eliminated the need to open the reaction tubes post-amplification. A large amount of amplicons are produced during the LAMP process and reducing the risk of releasing these amplicons into the testing environment is crucial for non-laboratory settings. For POC use, it is advantageous to be able to assess sample positivity immediately post-amplification, without any additional steps.

Further assay improvements included the design of a novel primer set that recognizes a conserved region within the HIV-1 integrase sequence. Although assay performance for the RT and INT primer sets was demonstrated separately for comparison purposes, the primer sets can be combined in a multiplex RT-LAMP reaction without altering the sensitivity of the reaction [16]. The APTIMA HIV-1 Qualitative Assay and the COBAS AmpliPrep / COBAS TaqMan HIV-1 Test V2.0 have both demonstrated that detection of clinical isolates may be facilitated by targeting two separate, conserved regions within the HIV genome because of the sequence diversity of HIV-1 [27, 28]. Furthermore, a multiplexed approach may be beneficial to circumvent potential mismatches due to resistance mutations induced by antiretroviral use. For POC use, the RT-LAMP assay may be performed using low-tech, portable heating devices, such as the ESEQuant Tube Scanner (Qiagen, Valencia, CA)[29] or non-instrumented nucleic acid (NINA) heaters [8]. Similar performance between these devices and traditional thermal cyclers has been demonstrated for the HIV-1 RT-LAMP assay [8]. In the current study, however, a thermal cycler was used as a matter of convenience for large-scale testing.

Because of the predominant use of rapid tests in non-laboratory settings, a representative rapid test was used as the primary basis for comparison to the RT-LAMP assay. The antibody-based Multispot HIV-1/HIV-2 Rapid Test was selected for comparison, given that it is currently used as a supplemental assay in the laboratory testing algorithm. It should be noted, however, that the Multispot is not currently Clinical Laboratory Improvement Amendments (CLIA)-waived for use at the POC. In seroconverting individuals, we demonstrated earlier detection (1–3 weeks) with the RT-LAMP assay as compared to the Multispot HIV-1/HIV-2 Rapid Test. Since viral RNA is detectable in the plasma up to 4 weeks prior to antibody, depending on the specific test [30], these results were not unexpected. Furthermore, studies have demonstrated that the Multispot and other FDA-approved rapid tests show significant weaknesses in detecting early HIV infection [31, 32].

Although the LAMP method is attractive for the development of a rapid POC NAAT, the HIV-1 RT-LAMP assay may also be useful in select laboratory settings, because of the quicker turnaround time and lower cost compared to current laboratory NAAT platforms. The HIV-1 RT-LAMP assay is estimated to cost 15-40X less per test compared to APTIMA, depending on the individual laboratory cost for purchasing the APTIMA kit. In relation to the viral load for each seroconversion panel member, RT-LAMP exhibited the expected limit of detection for the assay, which is approximately 10^4^ RNA copies/ mL. Additionally, RT-LAMP had a slight or no delay in time to detection compared to the current FDA-approved NAAT in the seroconverting individuals. The viral loads of all seroconverters included in this study exceeded the limit of detection for the assay shortly post first RNA-positive test result. Likewise, peak viral loads during acute HIV infection are estimated to be >100,000 RNA copies/ mL [33].

At present, the fourth-generation is the most sensitive of the laboratory immunoassay platforms, since it detects anti-HIV-1 IgG and IgM antibodies and HIV-1 p24 antigen. Rapid fourth-generation tests, such as the Determine HIV-1/2 Ag/Ab Combo (Alere, Orlando, FL) [24, 34, 35], will likely soon be CLIA-waived for use at the POC, but data suggest that it is not as sensitive for detection of acute infection as laboratory platforms [24, 36–43]. Although p24 antigen narrows the window from infection to detection, nucleic acid remains the earliest detectable biomarker for HIV infection [44]. In support of this, the RT-LAMP assay detected infection up to two weeks earlier than the GS HIV Combo Ag/Ab EIA.

One of the weaknesses of the RT-LAMP assay is that the current format is not as sensitive as the APTIMA HIV-1 RNA Qualitative Assay, the only FDA-approved NAAT for HIV-1 diagnosis. In the current study, the APTIMA detected infection up to five days earlier than the RT-LAMP assay. This is not surprising given the large discrepancy in input sample volume between the two assays. When taking into account the extraction process that occurred prior to adding sample to the reaction, the RT-LAMP assay used only 7% of the sample volume required for APTIMA. It has been demonstrated that the sensitivity of the RT-LAMP assay can be increased by increasing the overall reaction volume [16]. In addition, HIV nucleic acids can be concentrated directly from the clinical specimen through membrane capture/ concentration methods [18, 45].

Ideally, the HIV-1 RT-LAMP assay would be used with whole blood obtained from a finger stick. In the current study, we demonstrated the assay performance with plasma samples, given the difficulty in obtaining well-characterized, longitudinal whole blood samples from recent seroconverters. Alternatively, commercial seroconversion panels with preexisting HIV testing data are readily available and can be used to assess the timing of test positivity relative to each other. It is not anticipated that the assay results will differ significantly with whole blood, since the performance of the HIV-1 RT-LAMP assay has been demonstrated in a previous study [8]. Ongoing studies involve the evaluation of single-use, disposable heating devices with lyophilized RT-LAMP reagents for detection of HIV from whole blood specimens. Another limitation of the current study is that the assay was specifically designed for detecting subtype B HIV infections, since the primary focus of our laboratory is detection of HIV in the US. For global use, a group M-conserved RT-LAMP assay is preferred. Parallel evaluations of a subtype-conserved RT-LAMP assay are being performed in our laboratory.

In summary, the availability of a rapid NAAT, such as the HIV-1 RT-LAMP assay, could improve the diagnosis of acutely infected individuals who might otherwise be missed by current rapid antibody tests. The HIV-1 RT-LAMP assay has the potential to be implemented at the POC, as a supplemental or confirmatory test, where NAAT testing is currently not feasible due to cost and time limitations.
